# Rezidivierende, schwere Epistaxis bei COVID-19-Patienten

**DOI:** 10.1007/s00112-022-01615-4

**Published:** 2022-10-07

**Authors:** R. Isberner, U. Vorwerk, D. Schewe

**Affiliations:** 1 Universitätsklinik für Kinder- und Jugendmedizin, Universitätskinderklinik Magdeburg, Leipziger Str. 44, 39120 Magdeburg, Deutschland; 2 Universitätsklinik für Hals‑, Nasen‑, Ohrenheilkunde Magdeburg, Leipziger Straße 44, 39120 Magdeburg, Deutschland

**Keywords:** Nasenbluten, SARS-CoV2-Infektion, Mukosainflammation, Hämorrhagie, Schock, Nosebleed, SARS-CoV2-infection, Mucosal inflammation, Hemorrhage, Shock

## Abstract

Eine 12-jährige Patientin stellte sich mit fulminantem Nasenbluten vor; nebenbefundlich hatte sie eine COVID-19-Infektion. Bei bestehender Hämorrhagie zeigte sie eine zunehmende Schocksymptomatik. In der aktuellen Literatur ist eine Assoziation des SARS-CoV-2-Virus zu neu auftretender schwerer Epistaxis beschrieben, am ehesten durch Induktion einer Mukosainflammation. Andere Ursachen müssen jedoch ausgeschlossen werden.

## Abstract (en)

A 12-year-old female patient presented in the emergency room due to spontaneous, recurrent, fulminant epistaxis. As an additional finding a COVID-19-infection was noticed. Persisting hemorrhage led to increasing symptoms of shock. The current literature indicates a clear correlation of a SARS-CoV‑2-infection to spontaneous fulminant epistaxis, most likely due to a mucosal inflammation; however, other typical causes must be ruled out.

Epistaxis ist ein häufiges Krankheitsbild im Kindesalter. Die Ursachen sind vielfältig. Neue Studien zu Patienten mit SARS-CoV-2-Infektionen zeigen eine erhöhte Rate des Auftretens von Epistaxis. Die Datenlage in der Pädiatrie ist kaum beschrieben.

## Falldarstellung

### Anamnese und Befunde

Ein 12-jähriges Mädchen stellte sich mit spontan rezidivierendem Nasenbluten in der Notfallambulanz vor. Sie war blass, Hämatome, Petechien, Schleimhautblutungen oder eine Hepatosplenomegalie bestanden nicht. Die Blutung wurde durch eine Tamponade versorgt. Nachdem die Patientin ca. 250 ml Hämatin erbrochen hatte und synkopiert war, erfolgte die Aufnahme auf die Intensivstation. Aufgrund einer positiven SARS-CoV-2-PCR („polymerase chain reaction“) musste die Patientin isoliert werden. Bei persistierender Blutung mit konsekutiver Anämie (Hämoglobin 3,8 mmol/l) verfiel die Patientin zunehmend in einen Schockzustand. Die Blutung musste operativ per Kauterisation gestillt werden. Am nächsten Tag fand bei refraktärer Sickerblutung die operative Revision statt. Insgesamt mussten im Verlauf 2 Erythrozytenkonzentrate transfundiert werden. Die Blutgasanalysen waren stets ausgeglichen. Das Blutbild zeigte eine Anämie sowie eine milde, das Nasenbluten nichterklärende Thrombozytopenie (min. 124 Gpt/l). Die Gerinnungsdiagnostik war unauffällig. Die Entzündungs- und Hämolyseparameter lagen im Normbereich.

### Diagnose

#### Rhinoskopie.

In der linken Nasenhöhle, am Ansatz der mittleren Nasenmuschel, ist ein hyperämischer Schleimhautbezirk (möglicherweise im Sinne einer Vaskulitis) aufgefallen.

#### Gerinnungslabor.

TPZ (Thromboplastinzeit), INR (International Normalized Ratio), aPTT (aktivierte partielle Thromboplastinzeit), Fibrinogen, D‑Dimer, Thrombinzeit, Antithrombin, vWF(von-Willebrand-Faktor)-Antigen, -Aktivität, Einzelfaktorenaktivität stets normwertig.

#### Curaçao-Kriterien.

Nur ein Kriterium ist erfüllt; die Diagnose eines M. Osler ist unwahrscheinlich [6].

#### CT mit Kontrastmittel.

**Figure 1:**
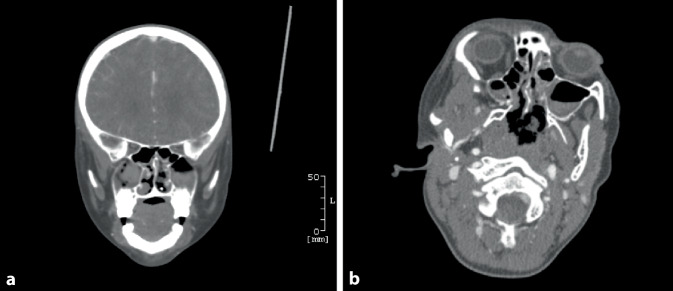

Hämatosinus in den Nasennebenhöhlen. Kein Hinweis auf eine Fraktur, Gefäßmalformationen, Fremdkörper oder malignes Geschehen **(**Abb. 1**)**.

### Therapie und Verlauf

Die Tamponaden konnten am 4. Tag nach der Reoperation entfernt werden. Die Patientin wurde nach 8 Tagen mit abschwellenden Nasentropfen bei Z. n. spontan rezidivierender Epistaxis aufgrund einer Vaskulitis, am ehesten durch die COVID19-Infektion, entlassen.

## Diskussion

### Epistaxis

**Figure 2:**
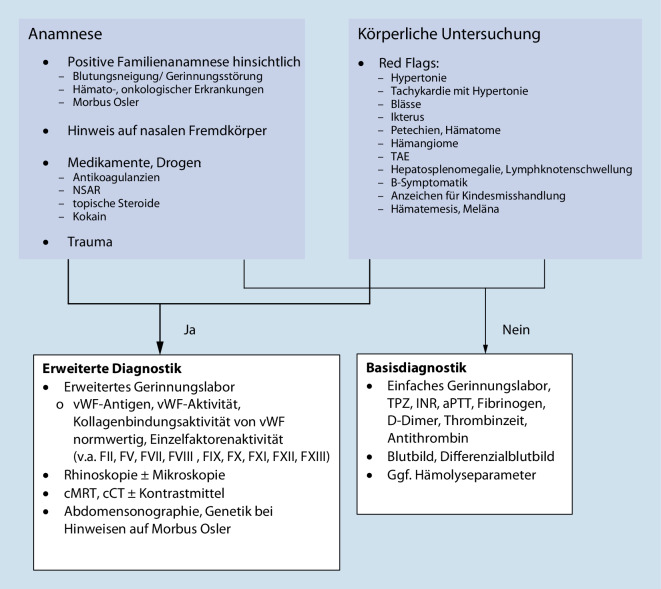

Nasenbluten tritt im Kindesalter häufig auf und verläuft, sofern die Quelle im Locus Kiesselbachi liegt, meist harmlos. Es gilt, lokale von systemischen Ursachen und deren klinische Wahrscheinlichkeiten zu unterscheiden (Tab. 1). Entzündliche Prozesse spielen eine große Rolle, v. a. bei allergischer Rhinitis und Infektionen der oberen Atemwege. Bei unauffälliger Anamnese und körperlicher Untersuchung mit Ausschluss von Red Flags wird eine basale Labordiagnostik empfohlen. Im Rahmen schwer stillbarer Blutungen sollten eine hämorrhagische Diathese, eine Hypertonie und auch Mittelgesichtsfrakturen als Ursachen ausgeschlossen werden. Diesbezüglich ist im Verlauf eine erweiterte Gerinnungsdiagnostik indiziert. In unserem Fall bestand weder anamnestisch, klinisch noch paraklinisch ein Anhalt für eine Gerinnungsstörung. Eine zerebrale Bildgebung (cMRT [craniale Magnet-Resonanz-Tomographie] bzw. cCT) ist je nach Anamnese zum Ausschluss von Frakturen, Gefäßmalformationen, Tumoren etc. durchzuführen (Abb. 2). Fehlbildungen und Raumforderungen waren aufgrund des schweren Verlaufes trotz operativer Versorgung nicht auszuschließen. Bei persistierender Blutung mit erneut notwendiger Erythrozytenkonzentrattransfusion wurde deshalb nach kritischer interdisziplinärer Diskussion (Pädiater, Radiologen, Hals-Nasen-Ohren-Heilkunde) die Indikation für eine cCT mit Kontrastmittel gestellt. Die ersten therapeutischen Maßnahmen bei persistierender Epistaxis umfassen eine Nasenflügelkompression und topische Vasokonstriktion z. B. mit Oxymetazolinhydrochlorid. Bei rezidivierendem oder persistierendem Verlauf einer Epistaxis sollte frühzeitig die Konsultation der HNO erfolgen, insbesondere bei unauffälliger Basisdiagnostik. In der Regel wird eine Rhinoskopie durchgeführt, mit Anlage einer Nasentamponade.

**Table 1:** 

Schleimhautläsion *(Häufigste Lokalisation im Locus Kiesselbachi)*	Infektionen, trockene Luft, allergische Rhinitis u.v.m
Trauma	Frakturen, nasale Intubation u.v.m
Tumoren	Juveniles nasopharyngeales Angiofibrom, lobuläres kapilläres Hämangiom u.v.m
Sonstige	Chronischer Husten (Mukoviszidose, Pertussis etc.) mit konsekutiv erhöhtem Nasenvenendruck
Hämorrhagische Diathesen	Hereditär/erworben, Thrombozytopathien/-penien, Koagulopathien, Vasopathien
Granulomatöse Erkrankungen	Granulomatöse Polyangiitis, Sarkoidose, Tuberkulose
Medikamente	Nichtsteroidale Antirheumatika, Antikoagulanzien u. v. m.

### Epistaxis und SARS-CoV-2

Spontane Epistaxis wird als ein klinischer Marker einer COVID-19-Infektion diskutiert [2, 4]. Daten über eine rezidivierende Epistaxis im Kindesalter i.R. einer SARS-CoV-2-Infektion fehlen.

Das SARS-CoV-2-Virus infiziert den Wirt über ACE2(„angiotensin-converting-enzyme 2“)-Rezeptoren, die u. a. durch Endothelzellen exprimiert werden [3]. Innerhalb des Respirationstraktes besitzt das nasale Endothel die höchste ACE2-Rezeptor-Dichte [7]. Die Rekrutierung von Immunzellen i.R. der Infektion kann mit einer Endotheldysfunktion und Apoptose einhergehen; konsekutiv kommt es zu einer mikrovaskulären Dysfunktion, Ischämie und Inflammation. Elektronenmikroskopisch kann die Vaskulitis der Nasenschleimhaut i.R. einer COVID-19-Infektion durch Viruspartikelnachweis von anderen entzündlichen Ätiologien unterschieden werden, was jedoch im klinischen Alltag nicht praktikabel erscheint [8]. Es wird postuliert, dass die Infektion mit der Delta-Variante mit Makroangiopathien und akuten Hämorrhagien einhergeht, wohingegen die anderen SARS-CoV-2-Varianten inflammatorische Mikroangiopathien mit thrombotischer Komponente verursachen. In einer pädiatrischen Kohorte konnten bei 72 von 76 Fällen die SARS-CoV-2-positiv getestet wurden, andere Ursachen einer gleichzeitig bestehenden Epistaxis ausgeschlossen werden [1]. Da dies auch in unserem Fall gilt, ist die COVID-19-Infektion als Ursache der vaskulitischen Veränderungen mit fulminantem Nasenbluten und beginnender Schocksymptomatik zu diskutieren.

## Fazit

- Bei spontaner, fulminanter oder rezidivierender Epistaxis ist eine SARS-CoV-2-PCR indiziert.
- Refraktäre Ätiologien gilt es auszuschließen. Auch ohne Gerinnungsstörung kann eine Epistaxis zum Schock führen (Red Flags beachten).
